# Population Analysis of Staphylococcus aureus Reveals a Cryptic, Highly Prevalent Superantigen SElW That Contributes to the Pathogenesis of Bacteremia

**DOI:** 10.1128/mBio.02082-20

**Published:** 2020-10-27

**Authors:** Manouk Vrieling, Stephen W. Tuffs, Gonzalo Yebra, Marleen Y. van Smoorenburg, Joana Alves, Amy C. Pickering, Joo Youn Park, Nogi Park, David E. Heinrichs, Lindert Benedictus, Timothy Connelley, Keun Seok Seo, John K. McCormick, J. Ross Fitzgerald

**Affiliations:** a The Roslin Institute, University of Edinburgh, Midlothian, United Kingdom; b Department of Microbiology and Immunology, University of Western Ontario, London, Ontario, Canada; c Department of Basic Sciences, College of Veterinary Medicine, Mississippi State University, Starkville, Mississippi, USA; d Lawson Health Research Institute, London, Ontario, Canada

**Keywords:** *Staphylococcus aureus*, T cells, evolution, pathogenesis, superantigens

## Abstract

Staphylococcal superantigens (SAgs) are a family of secreted toxins that stimulate T cell activation and are associated with an array of diseases in humans and livestock. Most SAgs produced by Staphylococcus aureus are encoded by mobile genetic elements, such as pathogenicity islands, bacteriophages, and plasmids, in a strain-dependent manner. Here, we carried out a population genomic analysis of >800 staphylococcal isolates representing the breadth of S. aureus diversity to investigate the distribution of all 26 identified SAg genes. Up to 14 SAg genes were identified per isolate with the most common gene *selw* (encoding a putative SAg, SElW) identified in 97% of isolates. Most isolates (62.5%) have a full-length open reading frame of *selw* with an alternative TTG start codon that may have precluded functional characterization of SElW to date. Here, we demonstrate that S. aureus uses the TTG start codon to translate a potent SAg SElW that induces Vβ-specific T cell proliferation, a defining feature of classical SAgs. SElW is the only SAg predicted to be expressed by isolates of the CC398 lineage, an important human and livestock epidemic clone. Deletion of *selw* in a representative CC398 clinical isolate, S. aureus NM001, resulted in complete loss of T cell mitogenicity *in vitro*, and *in vivo* expression of SElW by S. aureus increased the bacterial load in the liver during bloodstream infection of SAg-sensitive HLA-DR4 transgenic mice. Overall, we report the characterization of a novel, highly prevalent, and potent SAg that contributes to the pathogenesis of S. aureus infection.

## Abstract (precis)

Staphylococcus aureus is an important human and animal pathogen associated with an array of diseases, including life-threatening necrotizing pneumonia and infective endocarditis. The success of S. aureus as a pathogen has been linked in part to its ability to manipulate the host immune response through the secretion of toxins and immune evasion molecules. The staphylococcal superantigens (SAgs) have been studied for decades, but their role in S. aureus pathogenesis is not well understood, and an appreciation for how SAgs manipulate the host immune response to promote infection may be crucial for the development of novel intervention strategies. Here, we characterized a widely prevalent, previously cryptic, staphylococcal SAg, SElW, that contributes to the severity of S. aureus infections caused by an important epidemic clone of S. aureus CC398. Our findings add to the understanding of staphylococcal SAg diversity and function and provide new insights into the capacity of S. aureus to cause disease.

## INTRODUCTION

Staphylococcus aureus is an opportunistic pathogen of global importance that causes a broad array of diseases in humans and livestock (1, 2). The pathogenicity of S. aureus has been linked to the expression of a myriad of virulence factors that promote colonization, host immune evasion, and nutrient acquisition (3, 4). Staphylococcal superantigens (SAgs) are a family of at least 26 secreted toxins that modify the immune response by bypassing antigen processing and presentation and inducing activation of T lymphocytes. SAgs simultaneously bind major histocompatibility complex class II (MHC-II) molecules and T cell receptor Vβ-segments, leading to uncontrolled T cell proliferation and release of proinflammatory cytokines (5). SAgs are associated with the pathogenesis of a variety of diseases, including necrotizing pneumonia (6, 7), toxic shock syndrome (TSS) (8), food poisoning (9), and certain autoimmune diseases in humans (10, 11) and mastitis in dairy cows (12).

Almost all SAgs are encoded by mobile genetic elements (MGEs), such as plasmids, prophages, and staphylococcal pathogenicity islands (SaPIs) or variable genomic islands, and are distributed in a strain-dependent manner (5). In addition to the strain-variable SAgs, the core genome-encoded SAg SElX is carried by the great majority of S. aureus isolates and has been demonstrated to contribute to disease pathogenesis (7, 13, 14). Previously, Okamura and colleagues identified a gene (*selw*) encoding a putative SAg with 36% amino acid identity to staphylococcal enterotoxin A (SEA) (15), but disruptive mutations in the open reading frame and lack of an ATG start codon in many strains led to suggestions that the gene has limited functionality (16).

Here, we employ a combination of population genomic and functional analyses to investigate the diversity, distribution, functionality, and role in the pathogenesis of this novel SAg SElW. We report that *selw* is the most prevalent SAg gene across the S. aureus species and is identified in nearly all strains of S. aureus. SElW exhibits potent superantigenic activity that promotes the survival of a representative human CC398 strain in a humanized transgenic mouse model of bacteremia. Taken together, these data reveal a previously cryptic and potent SAg that contributes to the virulence of an important human- and livestock-associated clone of S. aureus.

## RESULTS

### Population analysis of SAg gene distribution reveals *selw* in the vast majority of strains.

**FIG 1:**
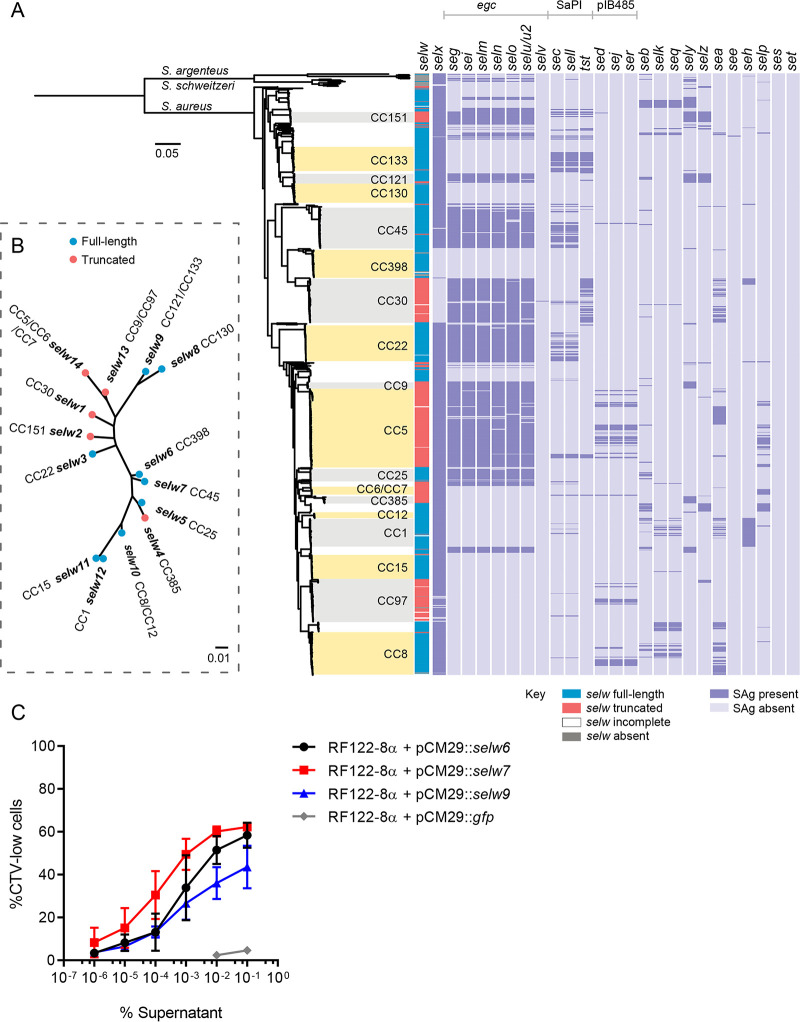
Population distribution, allelic variation, and T cell mitogenicity of *selw*. (A) Maximum likelihood tree constructed from a core genome SNP alignment of S. aureus (*n* = 786), *S. schweitzeri* (*n* = 10), and *S. argenteus* (*n* = 6) isolates depicting the repertoire of SAgs carried by each isolate. The left-most column indicates the presence of full-length (blue), truncated (red) *selw*, or incomplete *selw* due to assembly quality issues (blank); those cases lacking *selw* are gray. The remaining columns indicate the presence of all other SAgs in dark purple and absence in light purple. The major clonal lineages of S. aureus represented by ≥5 isolates in our database are indicated in the tree. (B) Maximum likelihood tree showing the phylogenetic relationship between the 14 major *selw* alleles. Full-length and truncated alleles are indicated with blue and red circles, respectively. (C) Proliferation of human PBMCs induced by supernatants of S. aureus RF122-8α containing *selw6*, *selw7*, or *selw9* in a pCM29 overexpression plasmid under the control of the *lukM* promoter. Supernatant from RF122-8α + pCM29::*gfp* was used as negative (vector) control. Proliferation levels were measured by assessing the loss of CellTrace Violet (CTV) staining by flow cytometry, and the percentage of CTV-low cells is plotted (mean ± SD from 3 separate donors).

We set out to examine the distribution and diversity of all known 26 SAg genes among 802 S. aureus complex isolates, including 786 S. aureus previously selected to represent the breadth of species diversity and host, geographical, and clinical associations, in addition to 6 Staphylococcus argenteus and 10 Staphylococcus schweitzeri isolates (17). A phylogeny of the isolates represented in our genome data set was reconstructed based on core genome single nucleotide polymorphism (SNP) variation, as described in the Materials and Methods, and the presence or absence of all SAg genes was determined and mapped onto the tree (Fig. 1). Overall, all isolates examined contained at least 1 and a maximum of 14 SAg genes (Fig. 1A). SAg genes not associated with discrete MGE, including genes *sely* and *selz* and members of the *egc* locus, were present in a largely lineage-dependent manner (12, 18), whereas those contained on MGE, such as the phage-encoded *sea* and plasmid pIB485-encoded *sed*, were more unevenly distributed across lineages consistent with horizontal gene transfer (Fig. 1A) (4, 5). Of note, human isolates carry on average more SAgs than nonhuman isolates (6.54 versus 4.25, respectively; Student’s *t* test, *P* = 2 × 10^−16^), reflecting a higher *egc* element carriage (positive carriage defined as having at least 5 of the *egc* genes) of human isolates than that of animal isolates (54.7% versus 26.0%; chi-square test, *P* = 4.8 × 10^−15^). Importantly, our analysis establishes that every isolate of S. aureus carries at least one intact SAg gene, which is consistent with an important role for SAgs in an array of diseases of humans and animals.

Recently, a novel putative SAg gene, *selw*, was identified widely in a collection of 51 S. aureus isolates, but the gene was predicted to be intact only in 4 strains (16). Remarkably, taking into account the possibility of an alternative start codon, we identified the *selw* gene to be present in 97.1% of all S. aureus isolates, with 93 unique alleles and 14 major allelic variants identified (contained in ≥10 isolates each), sharing 92.3% amino acid identity. The *S. argenteus* and *S. schweitzeri* genomes in our data set also contained *selw*, albeit only in one isolate each (Fig. 1A). A phylogenetic tree based on the *selw* sequences indicates that *selw* evolution has progressed largely in a lineage-specific manner (Fig. 1B). Of the isolates containing *selw*, 62.5%, representing 17 of 25 major S. aureus clonal complexes and 9 major *selw* allelic variants, contained a full-length intact *selw* gene variant. A further 37.5% of isolates, representing 8 of 25 major S. aureus clonal lineages and 5 major *selw* allelic variants, contained a disrupted *selw* truncated by a premature stop codon located 95 to 199 amino acids downstream of the predicted signal peptide cleavage site. Of these 5 truncated gene alleles, 3 arose by distinct mutations, but *selw13* and *selw14* contained the same nonsense mutation that was likely shared via recombination of a 399-bp sequence (see Fig. S1A in the supplemental material).

10.1128/mBio.02082-20.1 FIG S1 Recombination events in *selw* and structural modeling of SElW variants. (A) Predicted recombination events in *selw* as determined by RDP4. Predicted breaking points and origin of imported sequences are indicated. (B) Amino acid alignment of postsignal peptide sequence of SElW6, SElW7, and SElW9 was constructed using Clustal Omega. Amino acid polymorphisms between SElW9 and SElW7 are highlighted in turquoise, and substitutions between SElW6 and SElW7 are highlighted in orange (*, conserved, :, highly conservative substitution; ., weakly conservative substitution). Zinc binding residues are highlighted in pink. (C) Theoretical structural model of the SElW7 T cell activation complex generated using Phyre2. Predicted structure of SELW is shown with superpositioned MHC class II from both the high-affinity, zinc-dependent SEH-HLA-DR1 structure (PBD: 1HXY) and the low-affinity SEA-HLA-DR1 structure (PBD: 1LO5), as well as the TCR Vbeta5.1 chain from the SEK-Vβ5.1 structure (PBD: 2NTS). The SElW7 structure is shown in grey, residues differing from SElW6 and SElW9 are indicated in orange and turquoise, respectively, and zinc binding residues are in purple. Download FIG S1, PDF file, 0.1 MB. Copyright © 2020 Vrieling et al. 2020 Vrieling et al. This content is distributed under the terms of the Creative Commons Attribution 4.0 International license.

The presence or absence of *selw* was not host specific, as intact and truncated alleles were represented among both human- and animal-associated clones. The most frequent combination of intact SAg genes (55.1% of isolates) was the core genome-encoded *selx* and *selw* with or without other SAg genes, of which 31.2% contained both *selx* and *selw* without additional SAgs (Fig. 1). Notably, CC398, a major human- and livestock-associated clonal lineage, contained only *selw*, suggesting that this may be the only SAg expressed by this lineage. Of note, genomic island- or MGE-encoded SAg genes were more frequently found in isolates with a truncated *selw* than in those with an intact *selw*, namely, the *egc* cluster (∼66.2% versus ∼32.2%; chi-square test, *P* < 0.001), the pIB485-like plasmid containing *sed*, *sej*, *ser* (∼12.9% versus ∼2.5%; *P* < 0.001), and the SaPI-encoded *selp* (12.9% versus 3.6%; *P* = 0.02). In each case, the genomic island or MGE contained a SAg gene from the same phylogenetic group as *selw*, suggesting a possible functional compensation for the loss of SElW expression (group III) (5). Overall, we have demonstrated that *selw* is core genome encoded, contains considerable allelic variation, and the majority of isolates (62.5%) carry a full-length gene consistent with a functional SElW protein.

### SElW is mitogenic for human and bovine T cells in a Vβ-dependent manner.

*selw* has previously been proposed to be nonfunctional due to the lack of an ATG start codon and a truncated gene in some lineages (16). In the current study, we identified an alternative TTG start codon in frame with the *selw* gene. In order to examine if SElW can be translated by S. aureus from the TTG start codon, we cloned the *selw* genes from three phylogenetically distinct clonal lineages into overexpression plasmids containing the *lukM* promoter (19). Plasmids containing the *selw6* (CC398), *selw7* (CC45), and *selw9* (CC133) genes were transformed into the SAg-deficient S. aureus strain RF122-8, and the T cell mitogenic activity of the culture supernatant was determined (12). The supernatant from cultures of RF122-8α + pCM29::*selw6*, *selw7*, and *selw9* all induced T cell proliferation of human lymphocytes, suggesting that *selw* is a functional SAg with the ability to stimulate human T cells (Fig. 1C).

**FIG 2:**
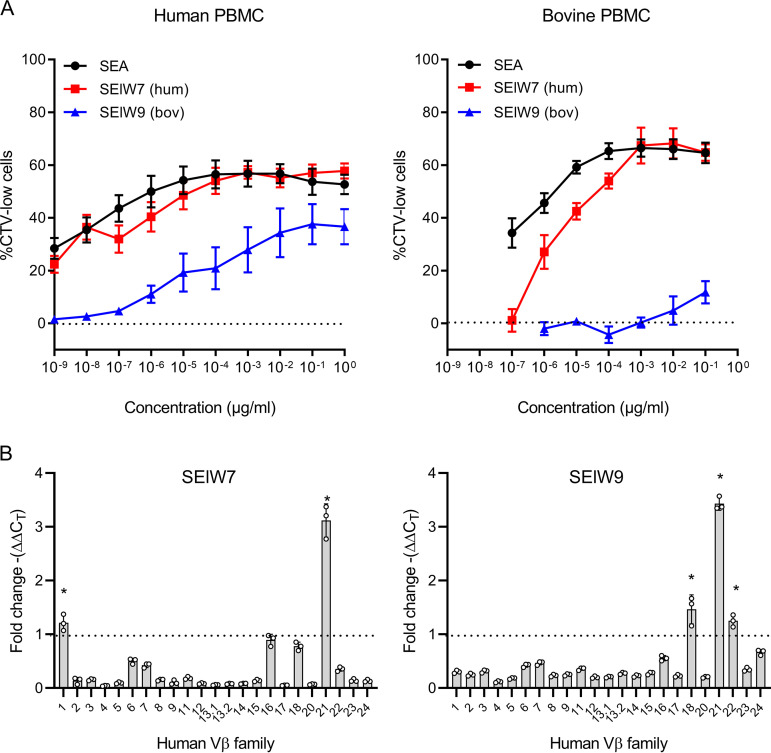
SElW activates human and bovine T cells and induces Vβ-specific T cell proliferation. (A) Analysis of SAg-induced proliferation levels by assessing loss of CellTrace Violet (CTV) staining using flow cytometry. Human and bovine PBMCs were stimulated with recombinant SAgs or buffer, and proliferation levels are indicated by the percentage of CTV-low cells. The percentage of CTV-low cells induced by SAg stimulation minus the percentage of CTV-low cells in buffer controls is plotted. Mean results ± SEM from 3 separate donors are shown. (B) Relative fold changes in human Vβ expression after stimulation with recombinant SElW7 and SElW9. Each dot represents an individual donor, and bars indicate means ± SD. Statistical differences were determined using a paired Student’s *t* test (*, *P < *0.05).

To further investigate the function and host specificity of *selw*, two distinct full-length allelic variants from human and ruminant isolates were selected for functional analysis, i.e., *selw7* from CC45 and *selw9* from CC133 which encode proteins with 91% amino acid identity (Fig. S1B). Recombinant SElW7 and SElW9 were analyzed for their ability to stimulate human and bovine peripheral blood mononuclear cells (PBMCs) in a proliferation assay compared to the previously characterized SEA (20) (Fig. 2A). As cattle contain high levels of γδ T cells in peripheral blood, we restricted our proliferation analysis to CD4^+^ T cells (21). Strikingly, the allelic variants differed considerably in their potency to induce T cell proliferation, with SElW7 stimulating human PBMCs to a similar level as the well-characterized, highly potent SEA, while SElW9 required >1,000-fold higher concentrations to reach the same proliferation level (Fig. 2A). For bovine PBMCs, both SELW7 and SEA stimulated bovine PBMCs in a dose-dependent manner, but SElW9 had a very limited response, suggesting that it has not evolved to stimulate bovine T cells.

Next, we evaluated the response of 23 human Vβ subfamilies to stimulation with SElW7 and SElW9 by real-time quantitative PCR (RT-qPCR) and found that SElW7 activated human Vβ 1 and 21, whereas SElW9 activated human Vβ 18, 21, and 22 (Fig. 2B). These data indicate that SElW is a classical SAg targeting Vβ subfamily 21 and that different variants of SElW can exhibit distinct Vβ-dependent T cell activation profiles (22). Structural modeling indicates that the most likely polymorphisms distinguishing the function of SElW7 and SElW9 are Tyr18 and Trp55, which are two residue side chains that would theoretically sandwich the CDR2 loop of the Vβ chain, a critical determinant for Vβ specificity (Fig. S1B and C) (23). From this model, there were no polymorphic residues that would directly alter binding to the MHC-II β-chain, and all 3 residues that likely bind zinc are conserved. Lys41 would be the only residue that could potentially alter the low-affinity MHC-II α-chain interface, but its location on the periphery of the interface suggests a limited impact (Fig. S1B and C).

### SElW is singularly responsible for the T cell mitogenic activity of the S. aureus human and livestock clone CC398.

**FIG 3:**
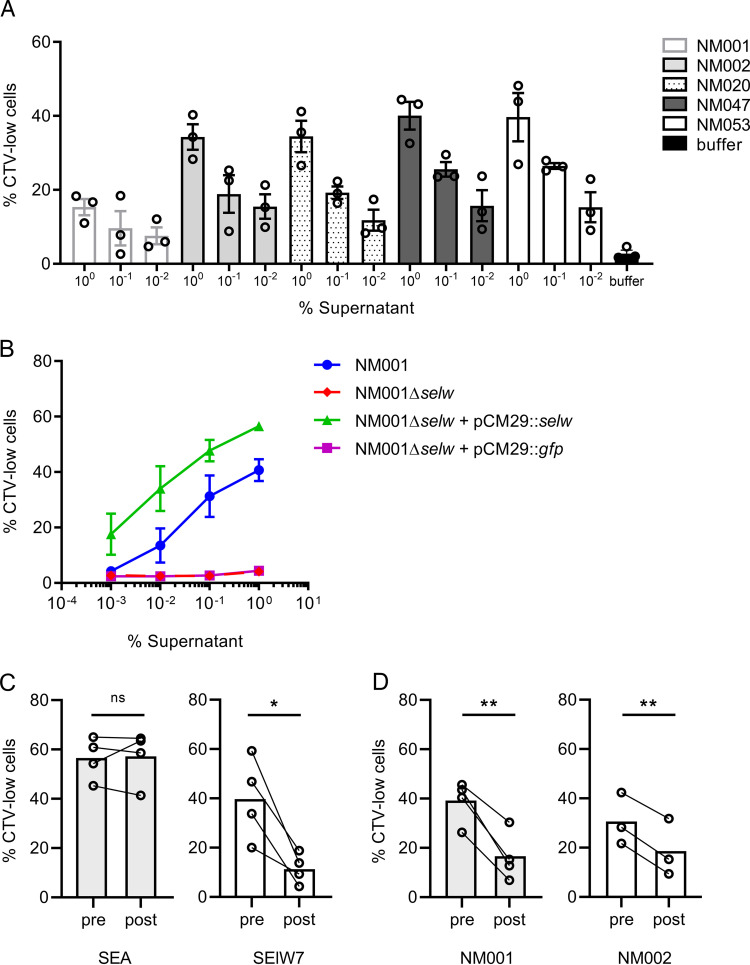
S. aureus CC398 NM001 requires SElW to stimulate human T cells. (A) Proliferation of human PBMCs stimulated with culture supernatant of representative CC398 isolates. Proliferation levels are shown as the percentage of CellTrace Violet (CTV)-low cells with each dot representing an individual donor and bars indicating mean ± SEM. (B) Proliferation of human PBMCs stimulated with culture supernatant of NM001, the isogenic Δ*selw* mutant (NM001Δ*selw*), the complemented mutant (NM001Δ*selw* + pCM29::*selw*), and a vector control (NM001Δ*selw* + pCM29::*gfp*). Mean percentages of CTV-low cells ± SEM from *n* = 3 separate donors are plotted. (C) Rabbit sera from animals immunized with recombinant SElW7 were analyzed for the ability to block mitogenicity of SElW. SAgs (1 × 10^−5 ^μg/ml) pretreated with 1% (vol/vol) preimmunization or 1% (vol/vol) postimmunization serum were incubated with human PBMCs, and T cell proliferation was analyzed by determining the percentage of CTV-low cells. A similar experiment was performed in D where 0.1% (vol/vol) supernatant of NM001 and NM002 pretreated with 3% (vol/vol) preimmunization or 3% (vol/vol) postimmunization serum was added to human PBMCs. Paired results from 3 to 4 individual donors are shown with bars representing the mean. Statistical differences were determined using a paired Student’s *t* test (*, *P < *0.05; **, *P < *0.01).

Having established that SElW is a potent SAg, we set out to examine the expression and the function of SElW among clinical S. aureus isolates. From our population genomic analysis of SAg gene distribution, we discovered that 31 of 37 CC398 isolates contained *selw* as their sole SAg gene (Fig. 1A). This lineage carries the *selw6* allele which differs from *selw7* in 4 positions in the amino acid sequence, of which none are predicted to affect binding to the TCR or MHC class II (Fig. S1B and C). In order to further investigate this gene association, we examined whole-genome sequences from 1,032 S. aureus ST398 revealing the presence of *selw* in 100% of isolates and additional SAg genes in only 2.8% of ST398 isolates, including *seb* (*n* = 19), *sea* (*n* = 4), or *selu/u2* (*n* = 2). These data confirm that *selw* is the only intact SAg gene in the vast majority of ST398 isolates, suggesting a key responsibility in S. aureus CC398 immune evasion. Accordingly, a panel of six CC398 strains from human infections containing only *selw* were selected for analysis in a T cell proliferation assay (24). Supernatants from strains NM001, NM002, NM008, NM020, NM047, and NM053 were incubated with human PBMCs at different concentrations and all were mitogenic for human PBMCs in a dose-dependent manner, indicating that CC398 isolates express a potent T cell mitogen (Fig. 3A). To investigate if SElW is completely responsible for the mitogenicity of S. aureus CC398, we generated an isogenic Δ*selw* mutant of isolate NM001 (NM001Δ*selw*) as described in the Materials Methods section. Whole-genome sequencing and culture experiments confirmed the deletion of *selw* and the absence of relevant off-target mutations and effects on growth *in vitro* (see Fig. S2 in the supplemental material). Culture supernatants of NM001 and NM001Δ*selw* were then incubated with human PBMCs, and deletion of *selw* resulted in the loss of capacity to stimulate T cell proliferation (Fig. 3B). Complementation of NM001Δ*selw* with pCM29*::selw* (containing *selw6* with its native promoter) restored the ability of NM001Δ*selw* to stimulate T cells, confirming that the mitogenic phenotype exhibited by ST398 NM001 is mediated by SElW. Next, we examined if SElW-specific antibodies could abrogate the mitogenic effects of SElW, on T cells, using polyclonal antibodies against SElW raised in rabbits and compared the effects of pre- and postimmunization serum on T cell stimulation by SElW. Immunization of rabbits with recombinant SElW yielded postimmunization serum that recognized SElW but not its closest homolog SEA (see Fig. S3A and B in the supplemental material). Importantly, incubation of recombinant SAgs with rabbit sera indicated that postimmunization serum neutralized the T cell mitogenicity of SElW but not SEA (Fig. 3C). Furthermore, postimmunization serum inhibited the mitogenic activity of supernatants of S. aureus ST398 strains NM001 and NM002 (Fig. 3D). Taken together, these data confirm that SElW is the sole secreted SAg of S. aureus CC398 and that the T cell mitogenicity of CC398 strains is SElW dependent.

10.1128/mBio.02082-20.2 FIG S2 Validation of NM001 mutants. (A) Whole-genome alignment of NM001, NM001Δ*selw*, NM001Δ*selw* + pCM29::*selw*, and NM001Δ*selw* + pCM29::*gfp* visualized with BLAST Ring Image Generator (BRIG). Deletion of *selw* was confirmed, and the following 2 nonsynonymous off-target SNPs were identified: one causing a His-to-Asp mutation in a “Transposase-associated ATP/GTP binding protein” and another Asn-to-Lys mutation in a “hypothetical protein.” (B) Growth analysis of NM001, NM001Δ*selw*, NM001Δ*selw* + pCM29::*selw*, and NM001Δ*selw* + pCM29::*gfp* in TSB. Mean results ± SD from 3 individual experiments are shown. Download FIG S2, PDF file, 0.8 MB. Copyright © 2020 Vrieling et al. 2020 Vrieling et al. This content is distributed under the terms of the Creative Commons Attribution 4.0 International license.

10.1128/mBio.02082-20.3 FIG S3 Validation of anti-SElW rabbit polyclonal antibodies and responses of wild-type and DR4 transgenic B6 mice to stimulation with SElW. (A) Western blot analysis of SAg proteins separated by SDS-PAGE and incubated with rabbit serum (1:2000) before (left) and after (right) immunization with SElW protein visualized by chemiluminescence. (B) Photograph of original Western blot shown in A and corresponding SDS-PAGE gel, including marker (Pageruler prestained protein ladder, 10 to 180 kDa; ThermoFisher Scientific). Lipase was included as additional negative control. (C) Splenocytes from B6 and DR4-B6 animals were incubated with SAgs, and IL-2 production was measured by ELISA. Means ± SEM are shown (*n* = 4 animals). Download FIG S3, PDF file, 0.1 MB. Copyright © 2020 Vrieling et al. 2020 Vrieling et al. This content is distributed under the terms of the Creative Commons Attribution 4.0 International license.

### SElW contributes to bacterial burden in the liver of mice infected with S. aureus.

**FIG 4:**
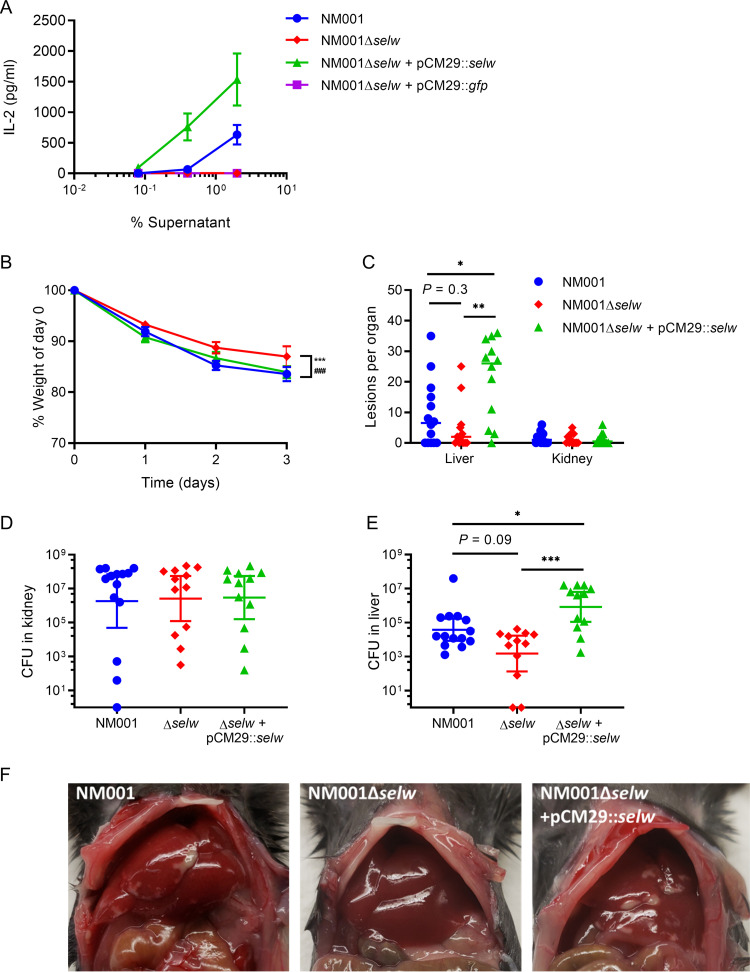
SElW promotes liver pathology and increased bacterial burden during S. aureus bacteremia. (A) Murine DR4-B6 splenocytes were incubated for 18 h with culture supernatant of NM001, NM001Δ*selw*, the complemented mutant NM001Δ*selw* + pCM29::*selw*, and a vector control NM001Δ*selw* + pCM29::*gfp*, and IL-2 production was measured by ELISA. Data are presented as the means ± SEM from *n* = 6 separate donors. (B) DR4-B6 mice were infected with NM001, *selw* mutants and were weighed daily, and weight loss was plotted (mean ± SEM with *n* = 12 to 14 mice per group). Differences between groups were determined by performing one-way analysis of variance (ANOVA) analysis on the area of each weight loss curve. For NM001 against NM001Δ*selw*, *** indicates a *P* value of <0.001; for NM001Δ*selw* + pCM29::*selw* against NM001Δ*selw*, ### indicates a *P* value of <0.001. (C) Endpoint was performed at day 3 postinfection, and surface lesions on kidneys and livers were counted. Each dot represents an individual mouse, and bars represent the median. *In vivo* bacterial burden of mice infected with S. aureus NM001 and mutants in kidneys (D) and liver (E) at 72 h are shown. Each dot represents an individual mouse, and the bar represents the geometric mean for CFU/organ ± 95% confidence interval. Statistical differences were determined using by the Kruskal-Wallis test (*, *P < *0.05; **, *P < *0.01; ***, *P < *0.001). (F) Representative liver pathology from infected mice.

To explore the suitability of a murine infection model for examining *in vivo* the role of SElW, we tested the potential of SElW for stimulating murine splenocytes. Splenocytes from HLA-DR4-IE humanized transgenic mice lacking endogenous mouse MHC-II on a C57BL/6 background (DR4-B6) (25) responded more strongly to recombinant SElW than splenocytes from wild-type C57BL/6 (B6) mice, as measured by interleukin-2 (IL-2) induction (Fig. S3C). IL-2 production was also elicited from DR4-B6 splenocytes by SElW-containing S. aureus supernatants and not the *selw* null mutant (Fig. 4A). Complementation of NM001*Δselw* with pCM29::*selw* produced culture supernatants that induced higher levels of proliferation of murine splenocytes than those of the wild-type strain as we had previously observed for human PBMCs (Fig. 3B, Fig. 4A). These data suggested that the DR4-B6 mouse represents an appropriate model for studying the *in vivo* effects of SElW, and accordingly, we intravenously inoculated mice with 1.5 × 10^7^ CFU of S. aureus NM001, NM001Δ*selw*, or NM001Δ*selw* + pCM29::*selw*, followed by monitoring of mice for 3 days and recording of weight loss (Fig. 4B). All three groups of mice lost between 10% and 15% of starting body weight, but the *selw* null mutant exhibited reduced weight loss (Fig. 4B). At endpoint, gross pathology on the livers and kidneys was assessed with relatively few lesions observed on the kidneys for all groups (Fig. 4C). However, the livers exhibited more severe tissue damage when infected with wild-type NM001 and the *selw*-complemented strain than with the *selw* null mutant (Fig. 4F). In addition, deletion of the *selw* gene resulted in a nonsignificant trend toward reduced lesion formation (*P* = 0.3) (Fig. 4C). However, there was a significant increase in lesion formation when mice were inoculated with the pCM29::*selw*-complemented mutant compared with both wild-type NM001 and NM001*Δselw* (Fig. 4C). Analysis of the bacterial burden in the kidneys reflected the gross pathology observations with no SElW-dependent differences in bacterial burden in this organ (Fig. 4D). In contrast, in the liver, we observed greater than a 1.5-log reduction in bacterial burden associated with the *selw* null mutant compared with wild-type NM001, representing a clear but nonsignificant trend (*P* = 0.09) (Fig. 4E). Furthermore, infections with the pCM29::*selw*-complemented mutant resulted in a strongly significant (nearly 3 log) increase in bacterial burden in the liver compared with infections with NM001*Δselw* (*P *= 0.0004) or the wild-type strain (*P *= 0.04) (Fig. 4E). These data indicate that the expression of SElW by S. aureus *in vivo* in a bacteremia model contributes to increased bacterial burden in the liver and that a higher level of expression corresponds with enhanced virulence (Fig. 4).

## DISCUSSION

Most SAgs are encoded by MGEs and are thus typically variable traits in clinical S. aureus strains. Here, we have characterized a novel, core genome-encoded, highly potent SAg, SElW. Previously, *selw* has been largely cryptic and overlooked as a putative SAg gene (16) for several reasons. First, its mis-annotation in S. aureus reference genomes suggested it was an allelic variant of *selu* (annotated as *selu2*) (26) or *sea* until Okumura and colleagues first named it *selw* (15). Second, *selw* does not have a classical ATG start codon, and we have demonstrated in the current study that the alternative TTG start site is functional. The only other functionally demonstrated example of a TTG start codon in S. aureus, to our knowledge, is staphylococcal protein A (SpA) (27, 28), and the atypical nature of the *selw* coding sequence has likely complicated attempts to identify and functionally characterize the translated protein. Third, the presence of a premature stop codon in *selw* in some S. aureus lineages has led to the suggestion that the gene may have limited functionality (12, 16). Of note, in the current study, truncated versions of SElW did not exhibit T cell proliferation activity (see Fig. S4 in the supplemental material).

10.1128/mBio.02082-20.4 FIG S4 Truncated SElW variants do not stimulate T cell proliferation. Proliferation of human PBMCs induced by supernatants of S. aureus RF122-8α containing *selw1*, *selw2*, *selw7_1-110*, *selw13*, and *selw14* in a pCM29 overexpression plasmid under the control of the *lukM* promoter. Supernatant from RF122-8α + pCM29::*selw7* was used as positive control and supernatant from RF122-8α + pCM29::*gfp* as negative (vector) control. Proliferation levels were measured by assessing the loss of CTV staining by flow cytometry, and the percentage of CTV-low cells is plotted for 2 individual donors. Download FIG S4, PDF file, 0.7 MB. Copyright © 2020 Vrieling et al. 2020 Vrieling et al. This content is distributed under the terms of the Creative Commons Attribution 4.0 International license.

The presence of the *selw* gene across the full breadth of S. aureus species diversity and its conserved genomic location adjacent to the *mntH* and *pfs* genes suggest an ancient acquisition by a progenitor of all extant S. aureus strains. Our analysis indicates that *selw* has diversified in a clonal lineage-specific manner with limited contribution of horizontal gene transfer to its evolution. Our functional analysis of different allelic variants from a ruminant and human lineage revealed a marked difference in SAg potency and Vβ-dependent T cell activation that was independent of the host species origin. Of note, some of the human Vβ subgroups targeted by SElW7 and SElW9 (Vβ18, Vβ21, and Vβ22 [Arden nomenclature; 29]) that correspond to bovine TRBV18, TRBV11, and TRBV2 (IMGT nomenclature [30]), respectively, are not functional in cattle (31). However, Vβ1 activated by SElW7 does have a functional bovine counterpart (TRBV9) (31), which likely explains why SElW7 is active on bovine cells while SElW9 is not. This information, along with the presence of truncated forms of *selw* in common bovine S. aureus clones CC97 and CC151, suggests the possibility of an alternative function or redundancy for SElW in cattle. Previous studies have demonstrated that variants of SElX, SElY, SElZ, and SEC from human, bovine, and ovine isolates differ in their potency to stimulate PBMCs from ruminant and human hosts (7, 12, 32, 33). While the SElW variants tested activated unique Vβ subsets, both strongly stimulated the Vβ21 subfamily of humans. Indeed, of the 26 SAgs identified to date, 10 SAgs target Vβ21, making it the most frequently targeted human Vβ by staphylococcal superantigens (5, 12), which is suggestive of structural features that allow Vβ21 to be more easily targeted. It is also feasible that Vβ21-expressing T cells play an important role in host defense against S. aureus infection and that targeting this T cell subset in particular promotes the survival of S. aureus during infection.

Our genomic analysis has found that up to 14 SAgs may be carried by a single strain and that the majority of clinical isolates of S. aureus contain more than 1 SAg gene (5, 18, 34). However, our population genomic analysis of >1,000 isolates of the important human- and livestock-associated S. aureus clonal lineage CC398 demonstrated that only a single full-length SAg, SElW, is carried by the vast majority (35–37). It has been reported previously that CC398 isolates contain a truncated variant of *selx* which is predicted to be nonfunctional (7). While pigs typically are colonized by S. aureus asymptomatically, CC398 can cause clinical disease in humans (24, 38), indicating that S. aureus strains that carry only SElW are pathogenic for humans. In support of this idea, we demonstrated that the expression of a single SAg SElW by a CC398 strain contributes to disease pathogenesis in the transgenic mouse model. Liver burden of the NM001 isolate was over 1.5 log higher than that of the *selw* null mutant, representing a nonsignificant trend suggestive of biological relevance. Importantly, when *selw* was encoded *in trans* (with its native promoter) and overexpressed, the bacterial burden increased significantly compared with both the null mutant and the NM001 wild type. Together, these data indicate that sufficient expression levels of SElW *in vivo* promote the pathogenicity of S. aureus. While splenocytes from HLA-transgenic mice are more sensitive to SAg activity than their C57BL/6 progenitors, they are not as responsive as human PBMCs (39). Added to this evidence, murine vasculature is not very representative of the human system and the vascular leakage reported in rabbit TSS models may not be as pronounced in mice. Therefore, we suggest that the impact of SElW observed in the murine model may be more pronounced during human infection.

Previously, use of the HLA-DR4 transgenic mouse model revealed that the liver is an important target of S. aureus-expressing SEA (40). Here, we demonstrate that SElW also promoted an increase in bacterial burden in the liver, which may reflect the organ’s importance as a key barrier that S. aureus must circumvent during bacteremia (41). Accordingly, SAgs, such as SElW, may be important in the establishment and persistence of bloodstream infections.

S. aureus is a leading cause of human and animal disease, and alternatives to antibiotics are urgently needed to limit the impact of antimicrobial resistance. The identification of a highly prevalent, core genome-encoded SAg that contributes to the pathogenesis of bacteremia, allied to the observation that its function can be neutralized by specific antibodies, suggests that SElW should be considered a component of a S. aureus vaccine targeting invasive infections.

## MATERIALS AND METHODS

### Ethics statement.

Written informed consent for drawing human venous blood was obtained from all healthy volunteers, recruited within the Roslin Institute (University of Edinburgh) or Mississippi State University. The use of human venous blood at the Roslin Institute was approved by the National Research Ethics Service (NRES) committee London City and East (reference 11/AL/0168). The use of human blood at Mississippi State University was in accordance with a human subject protocol (13-191) reviewed and approved by the institutional review board at Mississippi State University. Bovine blood was drawn from healthy Holstein-Friesian cows via the jugular vein under project license P803DD07A. This procedure was approved by the local ethics committee of the Roslin Institute in agreement with UK guidelines. All experiments were conducted according to relevant guidelines and regulations.

### Sequence analyses.

A whole-genome sequence data set of S. aureus (*n* = 786), *S. schweitzeri* (*n* = 10), and *S. argenteus* (*n* = 6) isolates representative of the global genetic and host species diversity of the S. aureus complex (17) was used to examine the distribution and diversity of all known SAg genes. In addition, a total of 1,032 available S. aureus sequence type 398 (ST398) sequences, which comprised assemblies deposited in GenBank (*n* = 633) and short read data in SRA (*n* = 399) selected by sequence type using Staphopia (42) were examined for SAg gene distribution. Illumina short-read sequence data were used to generate *de novo* assemblies using SPAdes v3.13 (43), which were annotated using Prokka v1.12 (44). A core SNP alignment was built using snippy and snippy-core v4.1 (https://github.com/tseemann/snippy), and a phylogenetic tree was constructed using FastTree v2.1.10 (45). The presence of *selw* and other previously described SAgs among the genome sequence data set was established by nucleotide BLAST (blastn) as implemented in blastable (https://github.com/bawee/blastable) using a threshold of 90% of identical positions to consider a gene present. All copies of the *selw* gene were extracted using blastn from the genome sequences above, aligned using MAFFT v7.313 (46), and a phylogenetic tree was constructed using FastTree v2 (45). Recombination analysis in the *selw* gene was performed using the Recombination Detection Program v4 (RDP4) (47). The pangenome of the whole-genome sequence data set was calculated with Roary v3.12 (48), and Scoary v1.6 (49) was used to identify genomes with either an intact or truncated *selw* gene.

### Bacterial strains and culture conditions.

S. aureus strains used in this study are listed in Table S1 in the supplemental material. All strains were cultured in tryptic soya broth (TSB) or brain heart infusion broth (BHI) with shaking at 200 rpm or on tryptic soya agar (TSA) plates at 37°C for 16 h unless stated otherwise. Where appropriate, media were supplemented with 10 μg/ml chloramphenicol. Culture supernatants were harvested by centrifugation and passed through a 0.45-μm filter and stored at −20°C. For a comparison of the growth of S. aureus mutants, overnight cultures were diluted in TSB until an optical density at 600 nm (OD_600_) of 0.05 and absorbance at OD_600_ was measured for 24 h using a Clariostar plate reader (BMG Labtech).

10.1128/mBio.02082-20.6 TABLE S1 S. aureus strains used in this study. Download Table S1, PDF file, 0.1 MB. Copyright © 2020 Vrieling et al. 2020 Vrieling et al. This content is distributed under the terms of the Creative Commons Attribution 4.0 International license.

### Recombinant protein expression and purification.

Recombinant SAg proteins were generated with a noncleavable N-terminal 6× His-tag in E. coli according to methods published previously (12). A detailed description is outlined in Text S1 in the supplemental material.

10.1128/mBio.02082-20.8 TEXT S1 Supplemental Materials and Methods. Download Text S1, PDF file, 0.2 MB. Copyright © 2020 Vrieling et al. 2020 Vrieling et al. This content is distributed under the terms of the Creative Commons Attribution 4.0 International license.

### Construction of pCM29::*selw*-containing strains.

SElW-expressing S. aureus strains were created as previously described, with a few modifications (50). In brief, SElW coding sequences were cloned into a pCM29 vector containing the active promoter of the leukocidin LukMF’ (19). To achieve this, pCM29::pLukM-sGFP (19) was digested with KpnI and EcoRI to remove the superfolder green fluorescent protein (sGFP) coding sequence while retaining the *lukM* promoter sequence. As digestion with these enzymes also removes the ribosomal binding site (RBS) from the plasmid, *selw* forward primers were designed to contain an RBS (51) upstream of the start codon, and *selw* sequences were amplified from genomic DNA of S. aureus strains CTH160 (*selw7*), DL643 (*selw9*), and NM001 (*selw6*) using the Q5 high-fidelity polymerase (New England BioLabs [NEB]) and primers listed in Table S2 in the supplemental material. Next, PCR products were assembled into pCM29 using Gibson assembly (NEB, UK) and subsequently transformed into heat shock-competent E. coli DC10B. Truncated SElW coding sequences were cloned into pCM29 under the *lukM* promoter using similar methods, and a truncated variant of *selw7* (*selw7_1-110*) was constructed by using a reverse primer introducing a stop codon at AA position 111 (primers in Table S2). For complementation of NM001*Δselw*, the pCM29::pLukM-sGFP vector (19) was digested with XbaI and EcoRI, removing both sGFP and the *lukM* promoter sequence. The *selw6* gene, including its own promoter sequence, was amplified from NM001 genomic DNA and assembled into pCM29 as described above. pCM29::*selw* plasmids were introduced into SAg-deficient S. aureus RF122-8α or NM001*Δselw* by electroporation.

10.1128/mBio.02082-20.7 TABLE S2 Primers used in this study. Download Table S2, PDF file, 0.02 MB. Copyright © 2020 Vrieling et al. 2020 Vrieling et al. This content is distributed under the terms of the Creative Commons Attribution 4.0 International license.

### Allelic replacement of *selw*.

Gene deletion constructs of *selw* were created in pJB38 (52). Plasmid construction and allelic replacement were performed as described previously (19). A detailed description of this protocol is outlined in Text S1.

### T cell proliferation assays.

For the collection of human and bovine venous blood, 10% acid-citrate-dextrose (ACD) was used as an anticoagulant. Peripheral blood mononuclear cells (PBMCs) were isolated from blood by density gradient centrifugation using Ficoll-Paque Plus medium (1.077 g/ml) (GE Healthcare, UK). Cells were cryopreserved at −155°C until further use. To assess proliferation, PBMCs were stained with CellTrace Violet at 1.75 μM (Invitrogen) in Hanks balanced salt solution (Gibco Life Technologies) for 10 min at room temperature (RT) and subsequently quenched using complete culture medium (RPMI 1640 (Sigma-Aldrich, UK) containing 10% (vol/vol) heat-inactivated fetal calf serum (FCS) (Gibco, UK), 200 nM GlutaMAX (Gibco), 100 U/ml penicillin, and 100 μg/ml streptomycin and also supplemented with 0.01% β-mercaptoethanol for bovine PMBCs. Next, cells were seeded in 96-well U-bottom plates at 4 × 10^5^ cells/well and stimulated with SAgs or culture supernatants for 5 days at 37°C with 5% CO_2_, unless stated otherwise. For SAg neutralization assays, SAgs or culture supernatants were incubated with pre- and postimmune serum from SElW7-immunized rabbits (Eurogentec, Belgium) for 30 min at 37°C prior to addition to the cells. At day 5, human PBMCs were treated with Live/Dead fixable yellow dead cell stain (ThermoFisher Scientific, USA), and the CTV staining of cells was measured by flow cytometry (BD LSRFortessa X20). Bovine PBMCs were stained with anti-CD4 (Clone IL-A12; IgG2a) and anti-δ TCR (Clone GB21A; IgG2b) primary antibodies and Alexa Fluor 647 anti-IgG2a (Southern Biotech, UK) and PE/Cy7-conjugated anti-IgG2b (ab130790; Abcam) secondary antibodies prior to flow cytometry. Data were analyzed using FlowJo v10.3 (Treestar), and the gating strategy is displayed in Fig. S5 in the supplemental material.

10.1128/mBio.02082-20.5 FIG S5 Flow cytometry gating strategy. (A) Human PBMCs with positive control (1 μg/ml SEA) and negative control (buffer). (B) Bovine PBMCs with positive control (0.1 μg/ml SEA) and negative control (buffer). Download FIG S5, PDF file, 1.8 MB. Copyright © 2020 Vrieling et al. 2020 Vrieling et al. This content is distributed under the terms of the Creative Commons Attribution 4.0 International license.

### Analysis of Vβ-dependent T-cell activation.

Human PBMCs (1 × 10^6^) were suspended in RPMI 1640 medium (Life Technologies) supplemented with 2% fetal bovine serum (FBS), 100 U/ml penicillin, and 100 μg/ml streptomycin. The cultures were stimulated with rSElW proteins (1 μg/ml) for 96 h at 37°C and 5% CO_2_. Total RNA was extracted from human PBMCs prior to and after stimulation with rSElW proteins, and cDNA was synthesized using a cDNA synthesis kit (Invitrogen). Selective expansion of human Vβ (huVβ) subfamilies was assessed by real-time quantitative PCR (RT-qPCR) using an ABI Prism 7500 real-time PCR system (Applied Biosystems, Foster City, CA) as described previously (53). Calculation to determine the percentage of each Vβ was determined by extrapolation of the threshold cycle to its standard curve as described previously (53). Selective expansion of each Vβ in the culture stimulated with SAg was determined when each %Vβ from the Sag-stimulated culture was divided by a corresponding %Vβ control (without stimulation). We used the Vβ subgroup nomenclature of Arden et al. (29).

### Mice.

Eight- to 11-week-old male and female HLA-DR4-IE (DRB1*0401) humanized transgenic mice lacking endogenous mouse MHC-II on a C57BL/6 (B6) background (here referred to as DR4-B6 mice) (25) were used for all *in vivo* infection experiments. Separately, male and female B6 and DR4-B6 mice retired from breeding purposes (approximately 40 weeks old) were used for splenocyte analysis experiments. All animal experiments were carried out in accordance with the Canadian Council on Animal Care Guide to the Care and Use of Experimental Animals, and the animal protocol was approved by the Animal Use Subcommittee at the University of Western Ontario.

### Murine cellular stimulation assay.

The ability of B6 and DR4-B6 mice to respond to SElW was determined using interleukin-2 (IL-2) production. Mouse spleens were collected and broken into a single-cell suspension, followed by erythrocyte lysis in ammonium-chloride-potassium (ACK) buffer. The remaining cells were suspended in RPMI (Invitrogen Life Technologies) supplemented with 10% FBS (Wisent Inc., Quebec, Canada), 100 μg/ml streptomycin, 100 U/ml penicillin (Gibco), 2 mM l-glutamine (Gibco), 1 mM MEM sodium pyruvate (Gibco), 100 μM nonessential amino acids (Gibco), 25 mM HEPES (pH 7.2), and 2 μg/ml polymyxin B (Gibco) and seeded into 96-well plates at a density of 1.1 × 10^6^ cells/ml. Titrating concentrations of recombinant SElW were added to cells and incubated for 18 h at 37°C with 5% CO_2_. Supernatants were assayed for IL-2 by enzyme-linked immunosorbent assay (ELISA) according to the manufacturer’s instructions (eBioscience). Supernatants from the S. aureus strains were tested for SAg activity using DR4-B6 splenocytes seeded into 96-well plates as described above. Titrations of supernatants from cultures of S. aureus NM001, NM001 Δ*selw*, and complemented mutant (grown in TSB for 4 h) were added to splenocytes for 18 h at 37°C with 5% CO_2_, and supernatants were assayed for IL-2 by ELISA.

### Staphylococcal bacteremia model.

Single bacterial colonies were picked from a TSA plate and grown in 3 ml TSB overnight (16 to 18 h). Cells were subsequently subcultured in TSB to an OD_600_ of 0.1, and grown to postexponential phase (OD_600_, ∼3.0 to 3.5). The bacterial pellet was washed once and resuspended in Hanks balanced salt solution (HBSS) to an OD_600_ of 0.125, corresponding to ∼ 1.5 × 10^8^ CFU/ml before injection of 1.5 × 10^7^ CFU of S. aureus in a total volume of 100 μl via the tail vein. Mice were weighed and monitored daily, sacrificed at 72 h postinfection, and the kidneys and liver aseptically harvested. All organs were homogenized, plated on mannitol salt agar (Difco) and incubated at 37°C overnight. S. aureus colonies were enumerated the following day with a limit of detection determined to be 3 CFU per 10 μl.

### Data analysis.

Statistical analysis was performed in Prism 8 (GraphPad, USA) and in R (R Foundation; https://www.R-project.org).
