# Supplementary figures and images for: Population Analysis of Staphylococcus aureus Reveals a Cryptic, Highly Prevalent Superantigen SElW That Contributes to the Pathogenesis of Bacteremia
Source: mBio. 2020 Oct 27;11(5):e02082-20. doi: 10.1128/mBio.02082-20 (PMC7593966; doi:10.1128/mBio.02082-20)

Figure S1

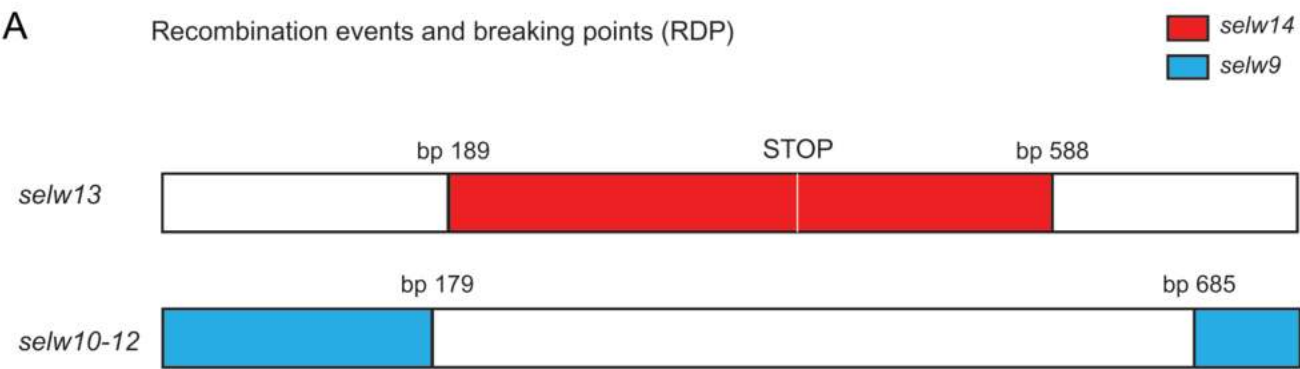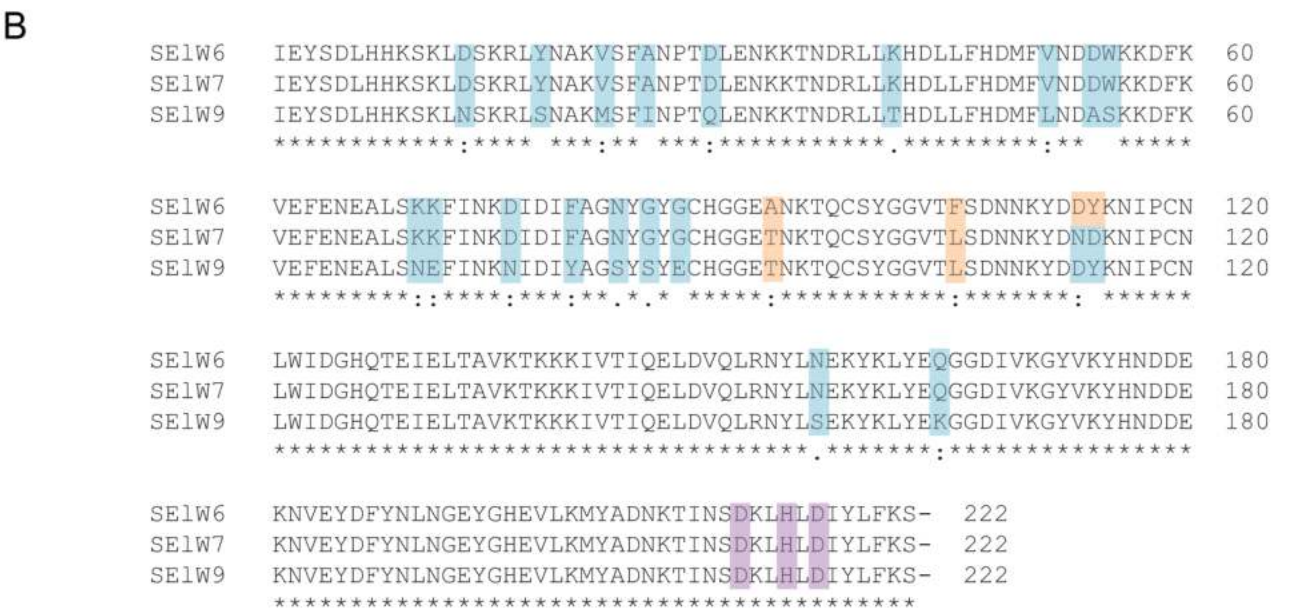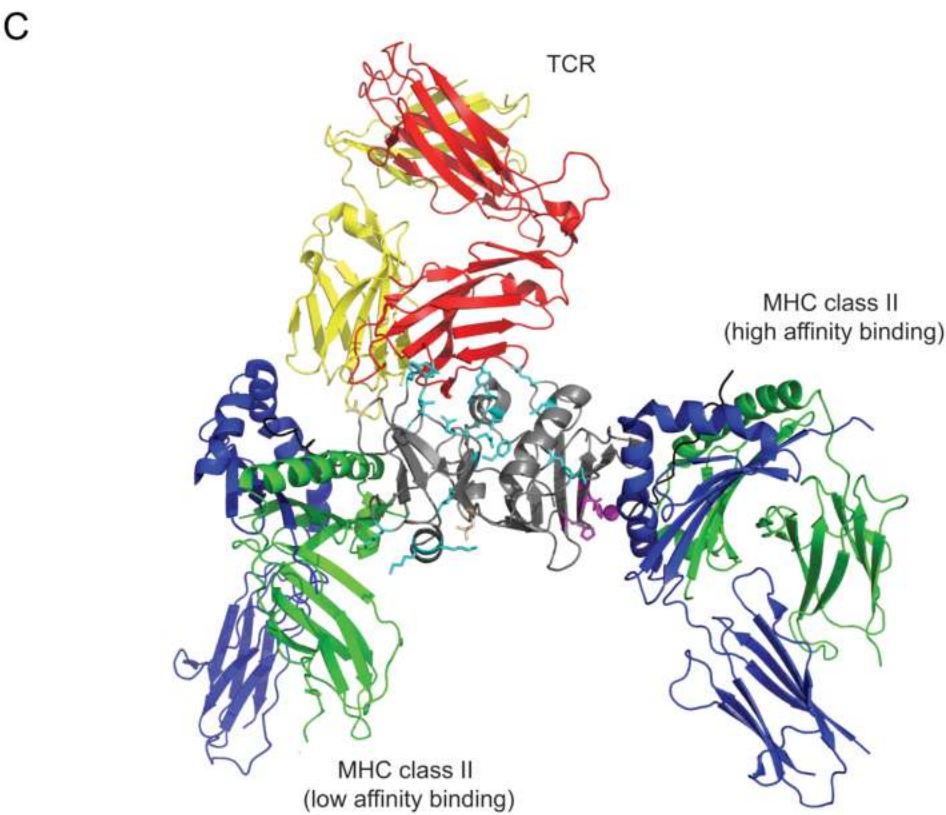

Supplement: FIG S1 [file mBio.02082-20-sf001.pdf]

Figure S2

A

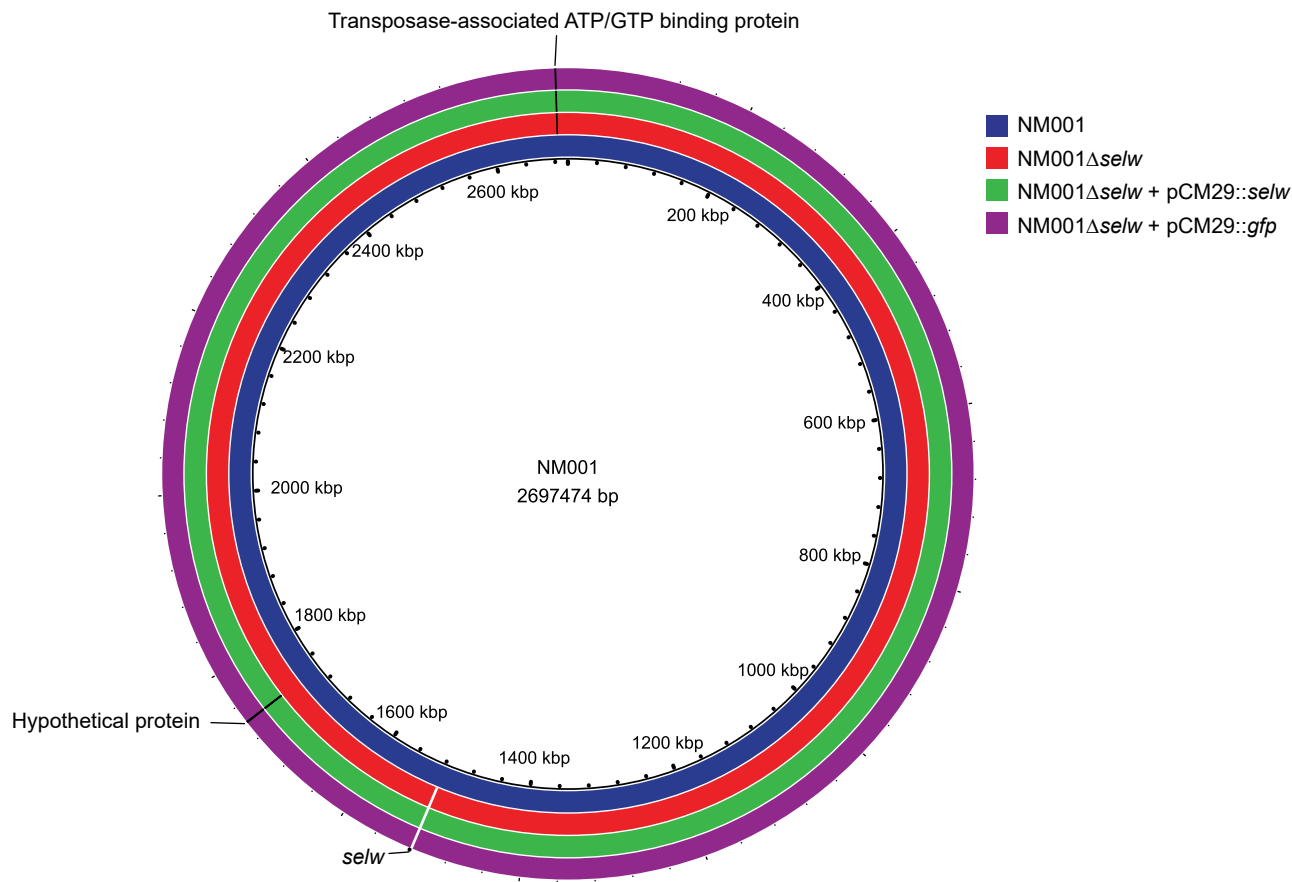

B

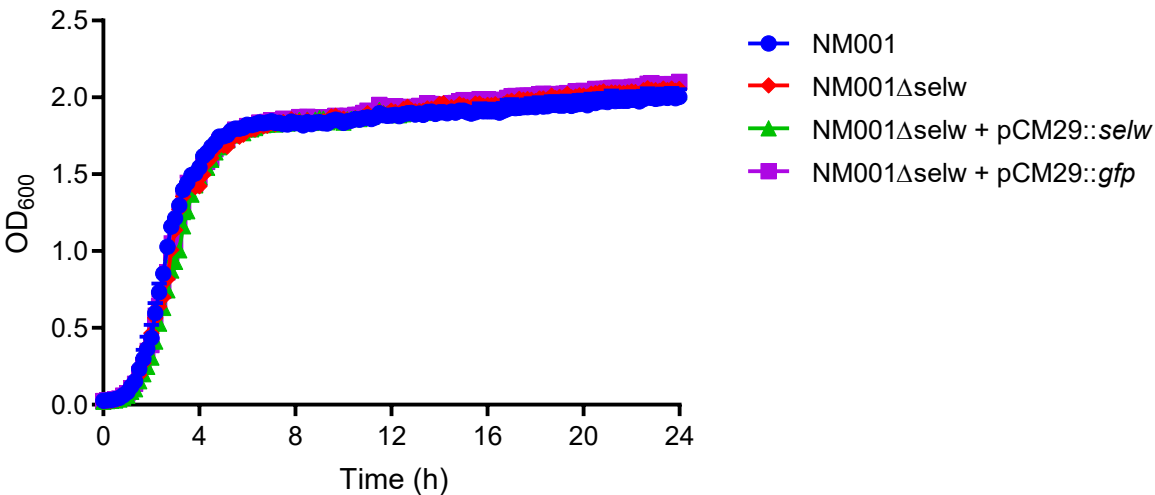

Supplement: FIG S2 [file mBio.02082-20-sf002.pdf]

Figure S3

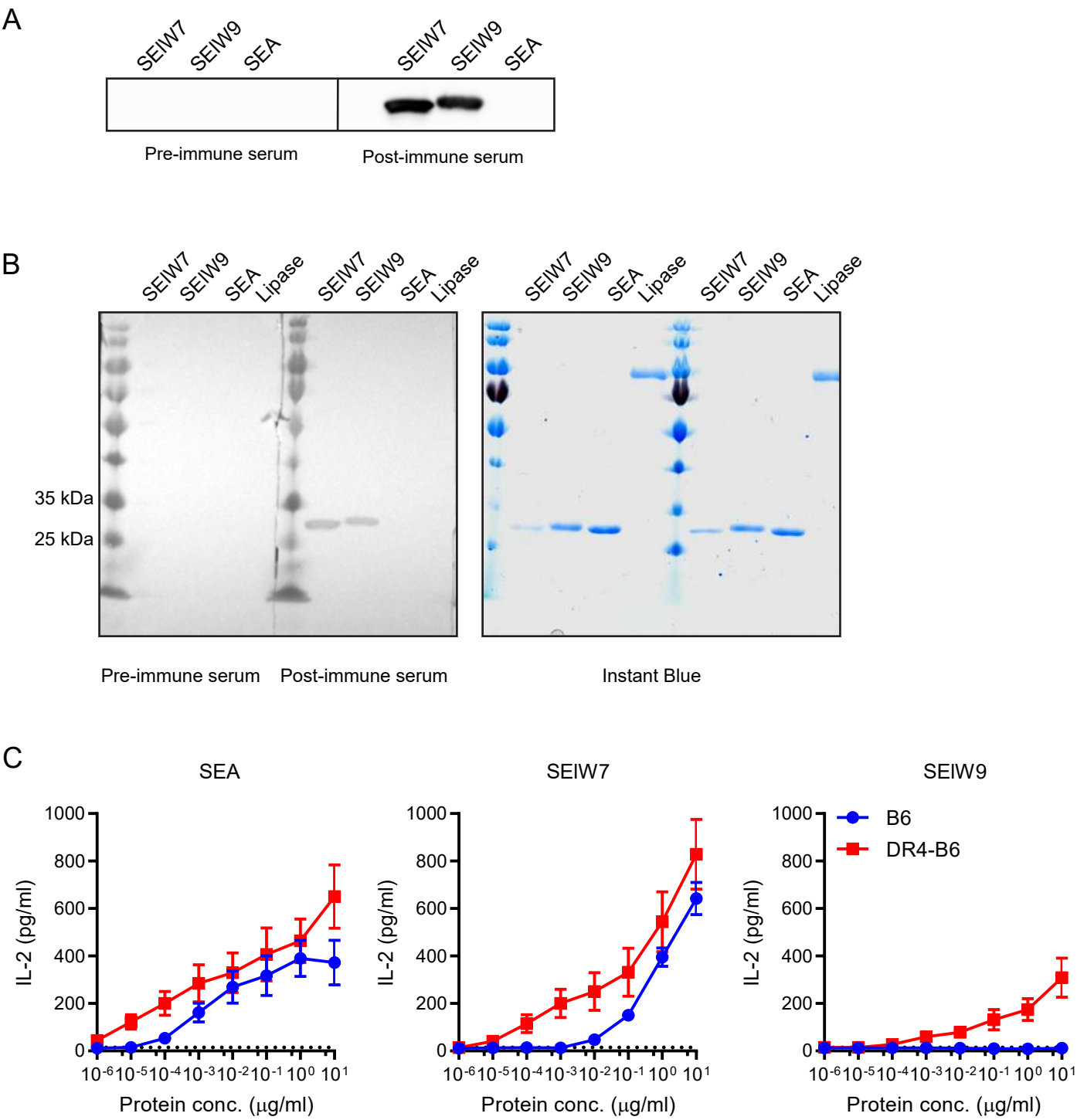

Supplement: FIG S3 [file mBio.02082-20-sf003.pdf]

Figure S4

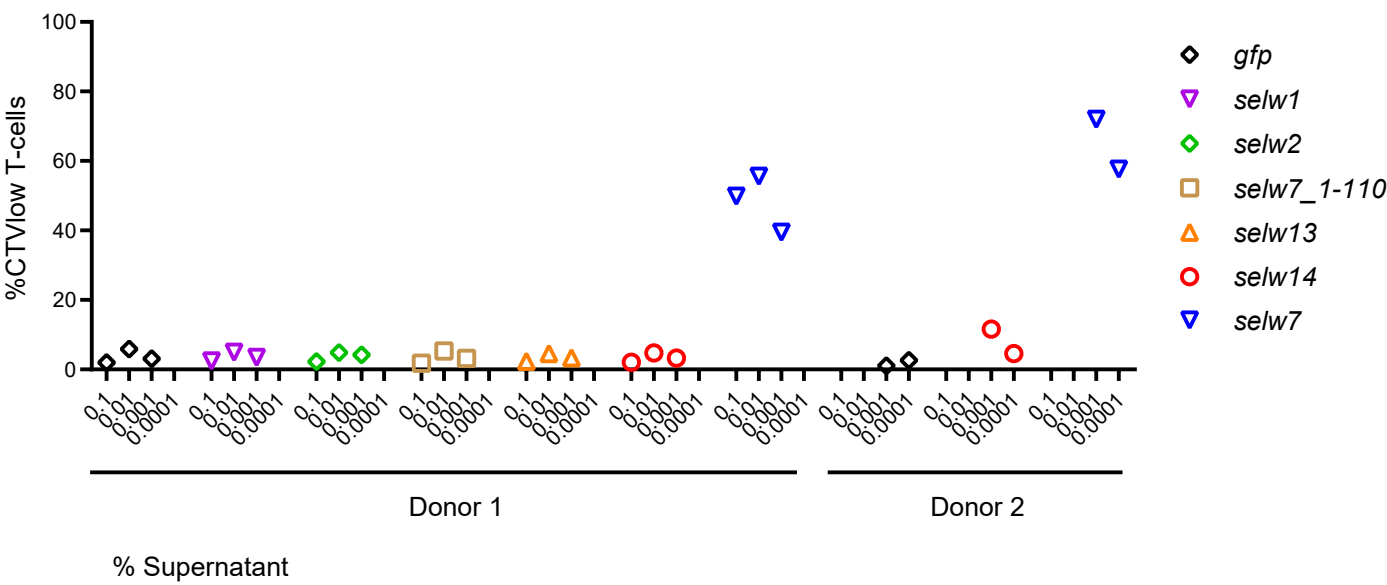

Supplement: FIG S4 [file mBio.02082-20-sf004.pdf]
