# Supplementary material for: Population Analysis of Staphylococcus aureus Reveals a Cryptic, Highly Prevalent Superantigen SElW That Contributes to the Pathogenesis of Bacteremia
Source: mBio. 2020 Oct 27;11(5):e02082-20. doi: 10.1128/mBio.02082-20 (PMC7593966; doi:10.1128/mBio.02082-20)
Supplement: TABLE S1 [file mBio.02082-20-st001.pdf]

Table S1: *S. aureus* strains used in this study

Strain	Description	Source/reference
CTH160	CC45 strain used to clone *selw7* from	(1)
DL643	CC121 strain used to clone *selw9* from	(1)
RF122-8α	SAg-deficient RF122, *Δhla*	(2)
RF122-8α + pCM29::*selw1*	RF122-8α containing *selw1* with LukM promotor	This study
RF122-8α + pCM29::*selw2*	RF122-8α containing *selw2* with LukM promotor	This study
RF122-8α + pCM29::*selw6*	RF122-8α containing *selw6* with LukM promotor	This study
RF122-8α + pCM29::*selw7*	RF122-8α containing *selw7* with LukM promotor	This study
RF122-8α + pCM29::*selw7_1-110*	RF122-8α containing *selw7_1-110* with LukM promotor	This study
RF122-8α + pCM29::*selw9*	RF122-8α containing *selw9* with LukM promotor	This study
RF122-8α + pCM29::*selw13*	RF122-8α containing *selw13* with LukM promotor	This study
RF122-8α + pCM29::*selw14*	RF122-8α containing *selw14* with LukM promotor	This study
RF122-8α + pCM29::*gfp*	RF122-8α containing *sgfp* with LukM promotor	This study
NM001	CC398 strain isolated from a human infection, Jul-07	(3)
NM002	CC398 strain isolated from a human infection, Jan-11	(3)
NM020	CC398 strain isolated from a human infection, Aug-11	(3)
NM047	CC398 strain isolated from a human infection, Jan-12	(3)
NM053	CC398 strain isolated from a human infection, Feb-10	(3)
NM001*Δselw*	Isogenic *selw* deletion mutant of NM001	This study
NM001*Δselw* + pCM29::*selw*	NM001*Δselw* complemented with pCM29::*selw* (*selw6* including native promotor)	This study
NM001*Δselw* + pCM29::*gfp*	NM001*Δselw* complemented with pCM29::*gfp*, vector control	This study

References.

1. Richardson EJ, Bacigalupe R, Harrison EM, Weinert LA, Lycett S, Vrieling M, Robb K, Hoskisson PA, Holden MTG, Feil EJ, Paterson GK, Tong SYC, Shittu A, van Wamel W, Aanensen DM, Parkhill J, Peacock SJ, Corander J, Holmes M, Fitzgerald JR. 2018. Gene exchange drives the ecological success of a multi-host bacterial pathogen. Nat Ecol Evol 2:1468-1478.
2. Wilson GJ, Tuffs SW, Wee BA, Seo KS, Park N, Connelley T, Guinane CM, Morrison WI, Fitzgerald JR. 2018. Bovine Staphylococcus aureus Superantigens Stimulate the Entire T Cell Repertoire of Cattle. Infect Immun 86:e00505-18.
3. Uhlemann AC, McAdam PR, Sullivan SB, Knox JR, Khiabanian H, Rabadan R, Davies PR, Fitzgerald JR, Lowy FD. 2017. Evolutionary Dynamics of Pandemic Methicillin-Sensitive Staphylococcus aureus ST398 and Its International Spread via Routes of Human Migration. mBio 8:e01375-16.
