# Supplementary material for: Population Analysis of Staphylococcus aureus Reveals a Cryptic, Highly Prevalent Superantigen SElW That Contributes to the Pathogenesis of Bacteremia
Source: mBio. 2020 Oct 27;11(5):e02082-20. doi: 10.1128/mBio.02082-20 (PMC7593966; doi:10.1128/mBio.02082-20)
Supplement: TEXT S1 [file mBio.02082-20-s0001.pdf]

Text S1: Supplemental Materials and Methods

Recombinant protein expression and purification. Genomic DNA was isolated from *S. aureus* strains CTH160 (for *selw7*), DL643 (for *selw9*) and Newman (for *sea*) using the bacterial genomic DNA purification kit (EdgeBio, UK). PCR of *selw* and *sea* genes was carried out with PfuUltra II Fusion HS DNA Polymerase (Agilent Technologies, UK) using a 5' primer annealing directly after the signal peptide cleavage site (as determined by SignalP 4.021) and a 3' primer that included the stop codon (Table S2). PCR products were cloned into pSC-B using the StrataClone PCR blunt cloning kit (Agilent Technologies, UK) according to the manufacturer's instructions. Plasmids were digested with BamHI and HindIII (*selw*), or BamHI and NdeI (*sea*), inserts separated and extracted after gel electrophoresis, followed by ligation into pQE-30 (QIAgen, UK) or pET15b (Invitrogen, Paisley, UK) using T4 DNA ligase (NEB, UK). Plasmids were subsequently transformed into competent *E. coli* XL-1 Blue or BL21 DE3 strains by electroporation and protein expression induced in mid-exponential growth phase (OD_{600} = 0.6) using 1mM isopropyl β-D-1-thiogalactopyranoside (IPTG) (ForMedium Ltd., UK) for 4 h at 37°C (SElW7 and SEA) or for 18 h at 16°C (SElW9) with shaking at 200 rpm. Proteins were released from *E. coli* using One Shot cell disruption (Constant Systems Ltd., UK) and purified by affinity chromatography with a HisTrap FF Crude column (GE Healthcare, UK) under native conditions. Elution was performed using an imidazole gradient and proteins were dialysed in PBS using a Spectra/Por Float-A-Lyzer (8-10 kDa MWCO) (Spectrum Laboratories, UK). Purity was analysed by SDS-PAGE and proteins were stored at -20°C.

Allelic replacement of *selw*. Gene deletion constructs of *selw* were created in pJB38 (1). In brief, the 1572 bp upstream region and the 759 bp downstream region of *selw* were amplified and spliced into pJB38 using Gibson Assembly (NEB, UK) using primers listed in Table S2. Plasmid pJB38 was introduced into *S. aureus* NM001 by electroporation and transformants were grown at replication-permissive temperature of 30°C in the presence of 10 µg/ml chloramphenicol. Plasmid integration was induced by growing transformants at the replication-restrictive temperature of 43°C. Single recombinants were diluted 1:1000 and sub-cultured a total of 5 times at 30°C to promote allelic exchange and plasmid excision. Finally, cultures were plated on counter-selection plates (200 ng/ml anhydrotetracycline) to select for clones that excised the plasmid (1-3). Double crossover mutants confirmed by PCR and sequencing were whole genome sequenced to check for any spurious mutations or errors (Microbes NG, Birmingham, UK).

Western immunoblot analysis. Recombinant SAgs (~0.7 µg/lane) were separated using SDS-page and transferred to a nitrocellulose membrane (Amersham Hybond ECL, GE Healthcare, UK). The membrane was incubated with blocking buffer (PBS containing 8% (wt/v) skimmed milk powder (Sigma-Aldrich, UK)) overnight at 4°C. The membrane was subsequently incubated with rabbit serum (Eurogentec, UK; 1:2000 unless stated otherwise) in PBST (PBS with 0.05% Tween 20 (Sigma Aldrich, UK)) containing 1% (wt/v) skimmed milk for 2h at RT. Next, the membrane was washed 3 times with PBST and incubated with Goat-anti-Rabbit-IgG-HRP (ab97051, Abcam) at 100 ng/ml for 1h at RT. The membrane was washed another three times and immunoreactivity was visualized by chemiluminescence using ECL (Amersham, GE Healthcare, UK).

References.

1. Bose JL, Fey PD, Bayles KW. 2013. Genetic tools to enhance the study of gene function and regulation in Staphylococcus aureus. Appl Environ Microbiol 79:2218-24.
2. Bae T, Schneewind O. 2006. Allelic replacement in Staphylococcus aureus with inducible counter-selection. Plasmid 55:58-63.
3. Vrieling M, Koymans KJ, Heesterbeek DA, Aerts PC, Rutten VP, de Haas CJ, van Kessel KP, Koets AP, Nijland R, van Strijp JA. 2015. Bovine Staphylococcus aureus Secretes the Leukocidin LukMF' To Kill Migrating Neutrophils through CCR1. MBio 6:e00335.
