# Supplementary material for: Population Analysis of Staphylococcus aureus Reveals a Cryptic, Highly Prevalent Superantigen SElW That Contributes to the Pathogenesis of Bacteremia
Source: mBio. 2020 Oct 27;11(5):e02082-20. doi: 10.1128/mBio.02082-20 (PMC7593966; doi:10.1128/mBio.02082-20)
Supplement: TABLE S2 [file mBio.02082-20-st002.pdf]

Table S2: Primers used in this study.

**Primer***	**Sequence (5' - 3')**
SElW Fw	CGGGATCCATCGAATATTCAGACTTACATC
SElW Rv	CCAAGCTTTTATGATTTGAATAAATAGATATCTAAATGC
SEA Fw	CCCCATATGAGCGAAAAAAGCGAAG
SEA Rv	CCCGGATCCTTAGCTGGTATACAGATAAAT
pQE-30 MCS Fw	CCCGAAAAGTGCCACCTG
pQE-30 MCS Rv	GTTCTGAGGTCATTACTGG
pCM29 *selw1* Fw	*GTATTAAAAATATAAAGGTACC*ttaggaggatgattatttTTGGGAGAGTTTGAAGTAAA
pCM29 *selw6* Fw	*GTATTAAAAATATAAAGGTACC*ttaggaggatgattatttTTGTGGGCTCACGAACCAC
pCM29 *selw7+13+14* Fw	*GTATTAAAAATATAAAGGTACC*ttaggaggatgattatttTTGGGAGAGTTTGAAGTTAA
pCM29 *selw2+9* Fw	*GTATTAAAAATATAAAGGTACC*ttaggaggatgattatttTTGATTTTGGGAGAGTTTG
pCM29 *selw* Fw	*TGCATGCCTGCAGGTCGACTCTAGA*ATGTAATATGTGATTCAGCTATAC
pCM29 *selw1* Rv	*GAAACAGCTATGACATGATTACGAATTC*TTACTTTTTCGTTTTCACTGC
pCM29 *selw2* Rv	*GAAACAGCTATGACATGATTACGAATTC*TTATTGGTTTCTACCACCATG
pCM29 *selw6+7+9* Rv	*GAAACAGCTATGACATGATTACGAATTC*TTATGATTTGAATAAATAGATATCTAAATGC
pCM29 *selw7_1-110* Rv	*GAAACAGCTATGACATGATTACGAATTC*TTACTTATTATTGTCACTTAAAGTAAC
pCM29 *selw13+14* Rv	*GAAACAGCTATGACATGATTACGAATTC*TTATTTATTATTGTCACTTAAAGTAACAC
pCM29 MCS Fw	GCATGTAACTGGGCAGTGTC
pCM29 MCS Rv	GTGAGCGGATAACAATTTCACAC
pJB38 *selw* UP Fw	**CACGAGGCCCTTTCGTCTTCAAGAATTC**CATTTTAAATGAGTAGAGGAGG
selw UP Rv	GATCTTTAATGATTAACTCACTTAAATTTTGATTATGTATCACGCTC
selw DN Fw	TTATGAGCGTGATACATAATCAAAATTTAAGTGAGTTAATCATTAAAG
pJB38 *selw* DN Rv	**GTCGACTCTAGAGGATCCCCGGGTACC**CCTAGCATTTGTTATTTTATC
pJB38 MCS Fw	CACCTGACGTCTAAGAAAC
pJB38 MCS Rv	AGGAGCGATTTACATATGAG
selw OUT Fw	ATTTAGCTAATCTAATAAAAATGG
selw OUT Rv	TTGTTACTTCTTCTTCCATG

**Fw and Rv refer to respectively forward and reverse primer. Restriction enzyme sites are underlined, pCM29 overlapping regions are shown in italics and the ribosomal binding site sequence is shown in lowercase. pJB38 overlapping regions are shown in bold.*
