# Supplementary material for: Population Analysis of Staphylococcus aureus Reveals a Cryptic, Highly Prevalent Superantigen SElW That Contributes to the Pathogenesis of Bacteremia
Source: mBio. 2020 Oct 27;11(5):e02082-20. doi: 10.1128/mBio.02082-20 (PMC7593966; doi:10.1128/mBio.02082-20)
Supplement: FIG S5 [file mBio.02082-20-sf005.pdf]

Figure S5

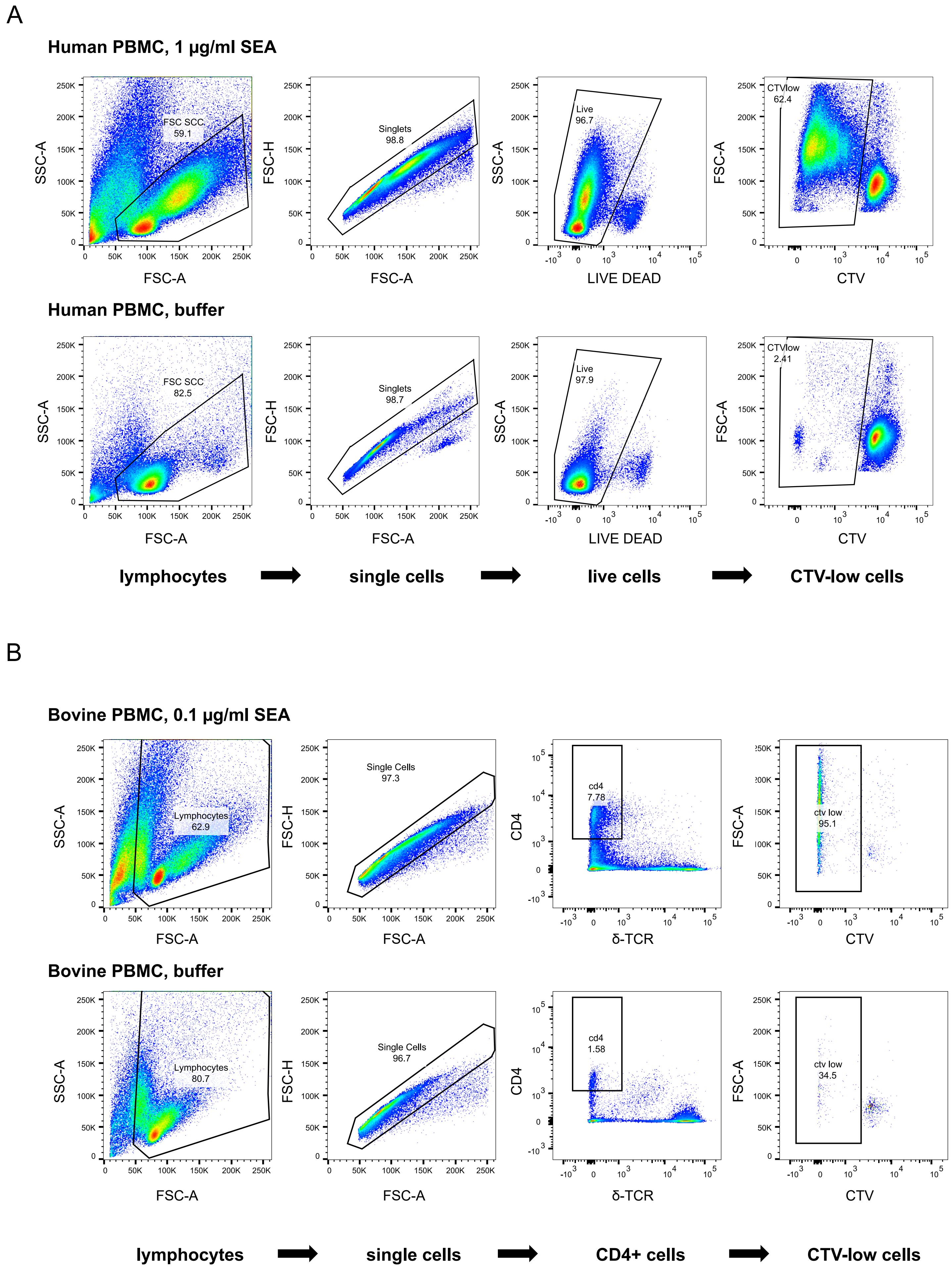

A
Human PBMC, 1 µg/ml SEA
FSC SCC
59.1
Singlets
98.8
Live
96.7
CTVlow
62.4
SSC-A
FSC-H
FSC-A
LIVE DEAD
CTV
Human PBMC, buffer
FSC SCC
82.5
Singlets
98.7
Live
97.9
CTVlow
2.41
lymphocytes
single cells
live cells
CTV-low cells
B
Bovine PBMC, 0.1 µg/ml SEA
Lymphocytes
62.9
Single Cells
97.3
cd4
7.78
ctv low
95.1
CD4
δ-TCR
Bovine PBMC, buffer
Lymphocytes
80.7
Single Cells
96.7
cd4
1.58
ctv low
34.5
lymphocytes
single cells
CD4+ cells
CTV-low cells
